# Accuracy of bony resection under computer-assisted navigation for bone sarcomas around the knee

**DOI:** 10.1186/s12957-023-03071-0

**Published:** 2023-06-21

**Authors:** Zhiping Deng, Qing Zhang, Lin Hao, Yi Ding, Xiaohui Niu, Weifeng Liu

**Affiliations:** 1 Department of Orthopaedic Oncology Surgery, Beijing Jishuitan Hospital, Beijing, 100035 China; 2 Department of Pathology, Beijing Jishuitan Hospital, Beijing, 100035 China

**Keywords:** Computer-assisted navigation, Bone sarcoma, Neoplasm, Oncology

## Abstract

**Background:**

Computer-assisted navigation has made bone sarcoma resections more precise. However, further clinical studies involving accuracy analyses under navigation are still warranted.

**Methods:**

A retrospective study for analysis of computer-assisted navigation accuracy was carried out. Between September 2008 and November 2017, 39 cases of bone sarcomas around the knee joint were resected under computer-assisted navigation. The control group comprised 117 cases of bone sarcomas around the knee treated by limb salvage surgery wherein bony cutting was achieved freehand. The length difference (LD) was defined as the specimen length minus the planned resection length. The LDs were detected in both groups and compared. The margin accuracy (MA) was defined as the achieved margin minus the desired margin at the bone cutting site and was detected in the navigation group.

**Results:**

The LDs between the postoperative specimen length and the preoperative planned length were compared. In the navigation group, the LD was 0.5 ± 2.5 mm (range, − 5 to 5 mm), while in the freehand group, the LD was 3.4 ± 9.6 mm (range, − 20 to 29 mm), with a significant difference (*P* < 0.01). In the absolute value analysis, the LD absolute value was 2.0 ± 1.6 mm in the navigation group and 8.3 ± 6.0 mm in the freehand group, with a significant difference (*P* < 0.01). In the navigation group, the MA was 0.3 ± 1.5 mm (range, − 3 to 3 mm) and the MA absolute value was 1.1 ± 1.0 mm.

**Conclusions:**

Better accuracy can be achieved when computer-assisted navigation is conducted for bone sarcoma resection around the knee.

**Level of evidence:**

IV.

## Highlights

- Better accuracy achieved when navigation is conducted for bone sarcoma resection
- The length difference (LD) was 0.5±2.5 mm (range, −5 to 5 mm) in navigation group
- The length difference (LD) was 3.4±9.6 mm (range, −20 to 29 mm) in control group
- The margin accuracy (MA) was 0.3±1.5 mm (range, −3 to 3 mm) in navigation group

## Introduction

Limb salvage surgery has been the mainstay of bone sarcoma treatments in recent years. Resection with a negative margin is the key for local control of malignant bone tumors [1]. Advances in imaging and computer-assisted technology have made tumor resections more precise. In pelvic and sacral tumor resections, computer-assisted navigation helped surgeons to reduce the intralesional margin rate [2–4]. In joint-sparing surgery, surgeons were able to obtain a clear margin under computer-assisted navigation [5, 6]. Furthermore, computer-assisted navigation was shown to have clinical benefits in other anatomical regions [7, 8]. The reasons why computer-assisted navigation can help bone tumor surgeons to obtain an adequate margin are the provision of real-time imaging on a computer and the improved accuracy of instruments during surgery. However, different tumor procedures have different accuracies for navigation. A few studies have focused on the accuracy of navigation in bone tumor models or cadavers [9–11]. However, clinical studies have usually included small numbers of cases for analysis of accuracy [12, 13]. Thus, further clinical studies for accuracy under navigation are still warranted. The distal femur and proximal tibia are the most common sites for primary bone sarcomas. The traditional method for bony resection around the knee is to measure the distance from an anatomical landmark during preoperative planning and then cut the bone freehand using a special instrument. The aim of the present study was to determine the accuracy of bony resection around the knee under computer-assisted navigation in our center. We included a freehand resection group of bone sarcomas around the knee as a control group.

## Materials and methods

This was a retrospective study. Between September 2008 and November 2017, 39 cases of bone sarcomas around the knee joint (29 in the distal femur; 10 in the proximal tibia) were resected under computer-assisted navigation (Stryker System) in our department by three groups of surgeons. The cases comprised 27 osteosarcomas, five chondrosarcomas, three spindle cell sarcomas, two adamantinomas, one undifferentiated pleomorphic sarcoma, and one Ewing sarcoma. All cases underwent intercalary resection in the femur or tibia. The reconstruction methods included intercalary custom-made prosthesis (*n* = 24), allograft (*n* = 11), and recycled frozen autograft (*n* = 4). In the early cases, the purpose of the navigation was to make the bony resection more precise and thereby match the custom-made prosthesis. Subsequently, the navigation was used when precise resection was required for sarcoma surgery around the knee. The clinical characteristics of patients were summarized in Table 1.

**Table 1:** Clinical characteristics of patients who underwent computer-aided intercalary resection (*n* = 39)

Characteristics	Values
Median age years	19 (8–60)
Gender	
Male	24
Female	15
Diagnose	
Osteosarcoma	27
Chondrosarcoma	5
Spindle cell sarcoma	3
Adamantinoma	2
Undifferentiated pleomorphic sarcoma	1
Ewing’s sarcoma	1
Tumor location	
Femur	29
Tibia	10
Staging	
Ib	2
IIb	36
III	1
Grade	
High	37
Low	2
Reconstruction methods	
Custom-made prosthesis	24
Allograft	11
Recycled frozen autograft	4

**Fig. 1:**
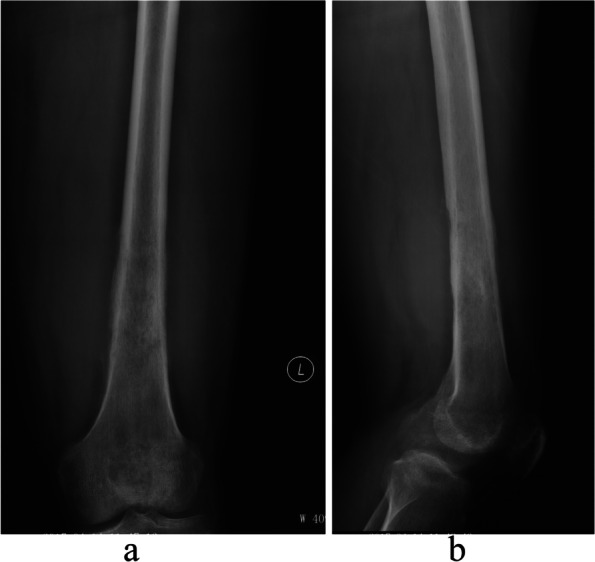
A 35 years old man, (**a**) The AP view. **b** The lateral view of distal femur

**Fig. 2:**
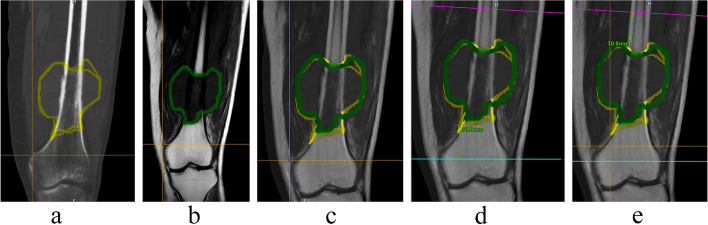
Preoperative planning on the navigation workstation. **a** CT and the tumor. **b** MRI and the tumor. **c** The fused image of CT and MRI with manually drawn tumor. **d** The designed margin for distal part with a plane on the navigation workstation. **e** The designed margin for proximal part with a plane

**Fig. 3:**
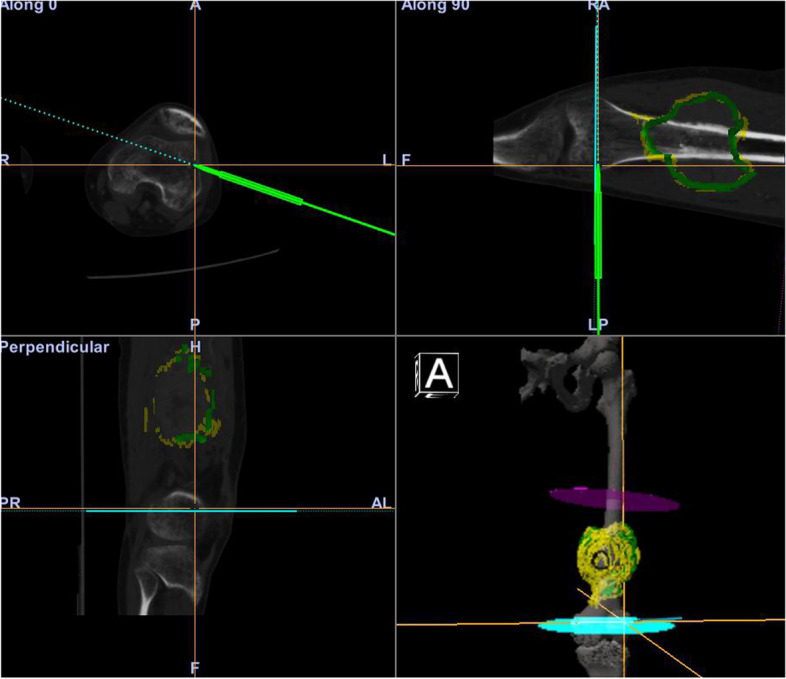
Computer-assisted navigation during surgery. The pointer is guiding the location and direction of bony cutting plane

**Fig. 4:**
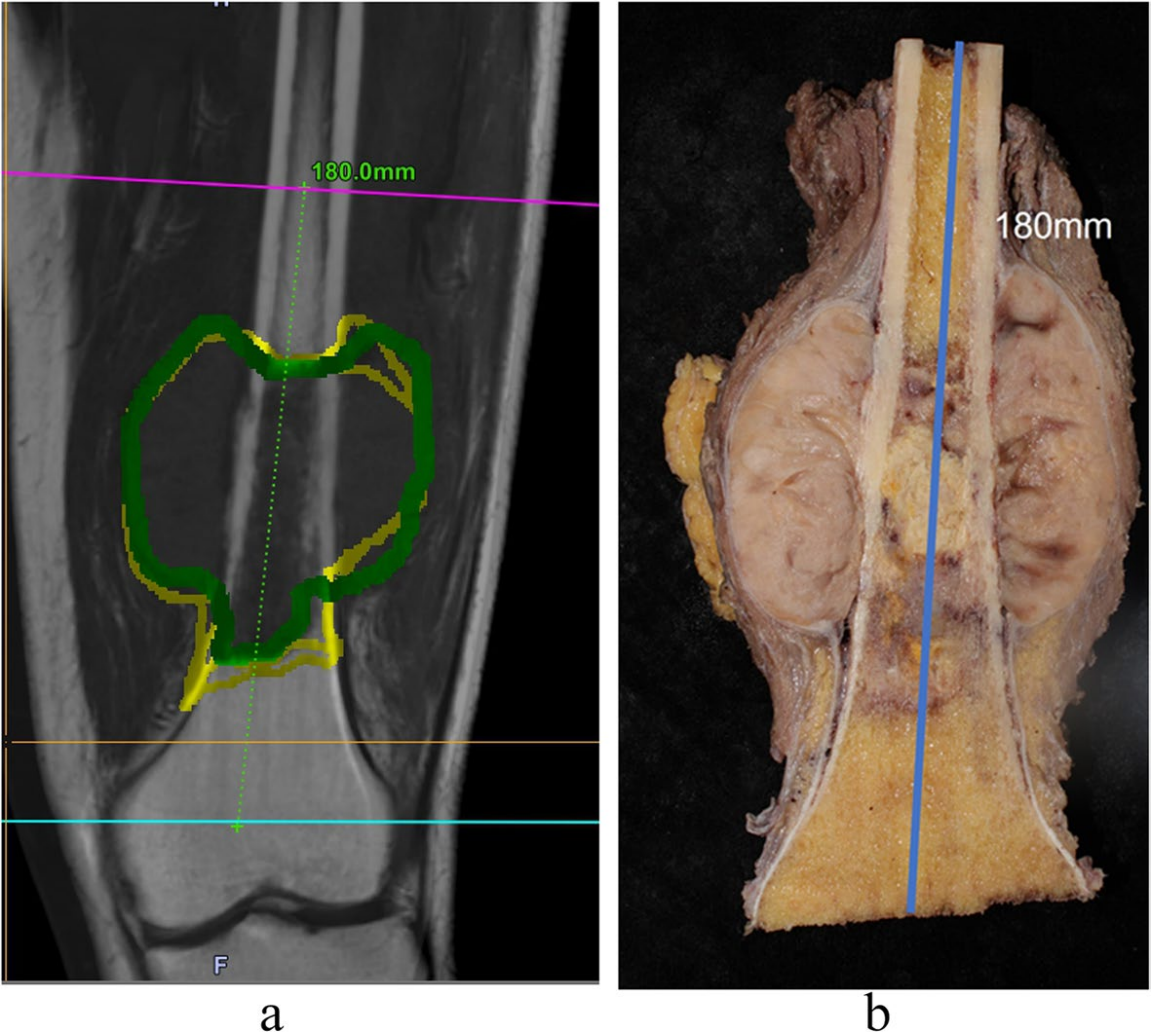
The correlation of preoperative planning and the specimen. **a** The preoperative planning. **b** The specimen

Preoperative planning was performed with OrthoMap software on a workstation (Stryker Company, Kalamazoo, MI). To show the tumor boundary better, pre-operative CT and MRI images were input into the system. The CT and MRI were fused, the tumor was drawn manually, and the surgical team determined the cutting plan regarding the distance in centimeters away from the tumor (Figs. 1 and 2). Wide margins were needed for this group of malignant tumors [1]. The desired distance varied according to the location. When the tumor was near the joint the desired margin was at least 1 cm and at the diaphyseal site the desired margin was 2–3 cm. The desired margin was recorded. The cutting planes were marked on the workstation. There were two types of registration methods. The first was the point-to-point method. We used six paired points for the anatomical landmark to finish the registration. The second was the imaging fusion method. During surgery, an Iso-C arm (Siemens, Henkestr. Erlangen, Germany) was used to obtain the bony image and then correlate the image to the previous CT and MRI images. After finishing the registration, we confirmed the cutting plane (Fig. 3), and three parallel Kirschner wires were inserted to the plane with the help of navigation while which was near the knee joint. Then we used a wire saw to cut the bone along the three Kirschner wires. The direction of the wire saw was consistent with the planning plane with this method. For the diaphyseal cutting plane, we used the wire saw to cut the bone when the location and direction were guided by navigation. The postoperative specimen was used for verification and compared with the preoperative plan (Fig. 4).

The length difference (LD) was defined as the specimen length minus the planned resection length. The bony margin was the nearest distance from the cutting edge to the tumor. The desired bony margin was planned by the surgical team and the achieved bony margin was measured by a musculoskeletal pathologist. The margin accuracy (MA) was defined as the achieved margin minus the desired margin at the bone cutting site. In recycled frozen autograft-treated cases, the margin could not be measured. Therefore, MA data were achieved in 35 patients, resulting in 70 values (two MAs in each case).

**Fig. 5:**
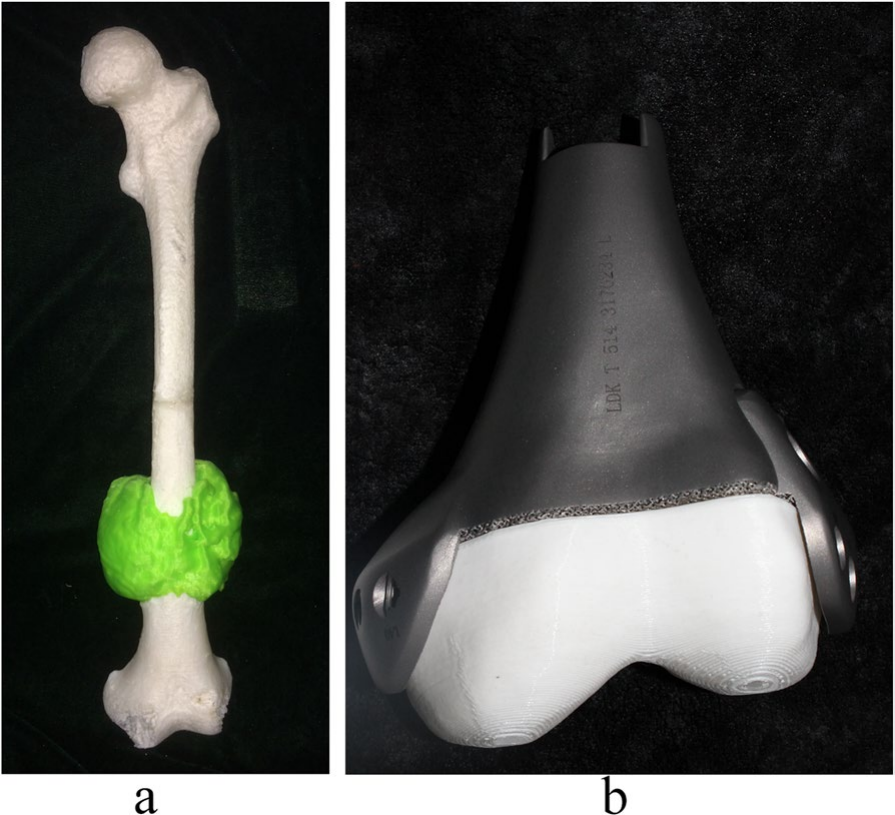
The 3D printed model was used during the surgical planning. **a** The model with tumor. **b** The matching of model and prosthesis

**Fig. 6:**
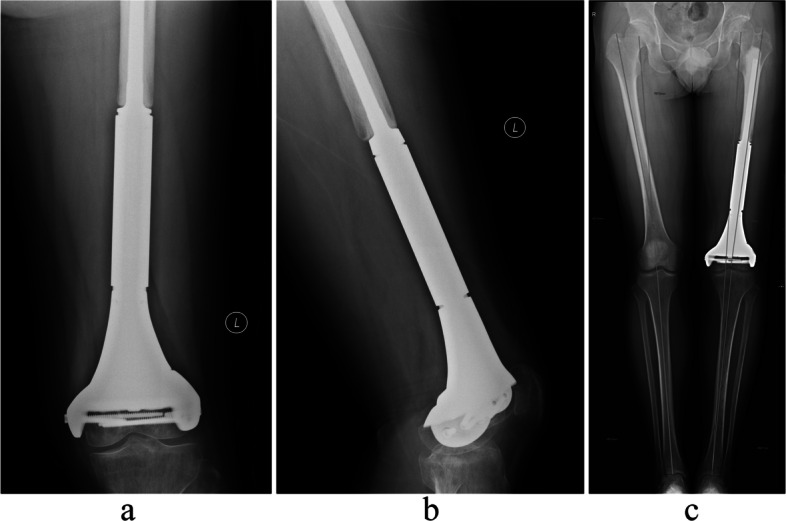
The post-operative imaging of this patient. **a** The AP view. **b** The lateral view. **c** The scanogram of lower limb

The custom-made prosthesis was used in twenty-four cases. The designed bony resection was one plane in one site, rather than multi-plane resection. The custom-made prosthesis was designed according to the resection plan. The 3D printed model was used during the surgical planning (Fig. 5). The conduct of navigation made the resection accurate and reconstruction matched well (Fig. 6).

**Fig. 7:**
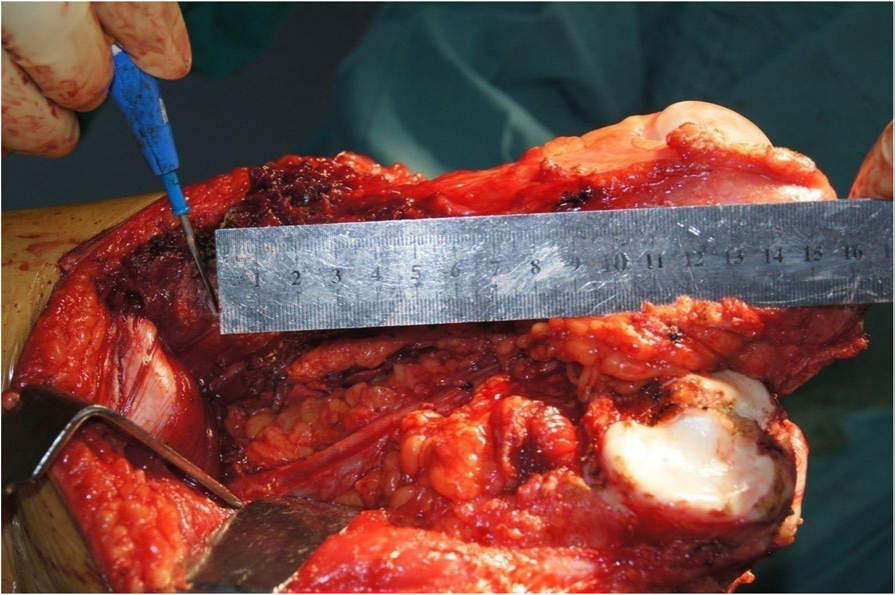
Intraoperative photo showing the method for using a ruler to measure the distance of the cutting plane to the anatomical landmark

The control group included 117 cases of bone sarcoma around the knee (87 in the distal femur; 30 in the proximal tibia) treated by limb salvage surgery wherein bony cutting was achieved freehand without computer-assisted navigation between May 2013 and January 2015. The characteristics of patients and the diameters of tumors were matched with the navigation group. All surgeries were performed by the same three groups of surgeons. Because we did not have a group of patients with the same intercalary resection without navigation, the surgery in the control group was tumor resection with only one bony cutting site. The other cutting site was at the knee joint. The method used for the determination of the cutting plane was to measure the length from the joint line to the planned plane with a ruler (Fig. 7). The instrument for cutting the bone was a wire saw, consistent with the navigation group. The length of the postoperative specimen obtained by freehand was compared with the surgical plan. The LD was detected in the control group. The differences between the two groups were compared and analyzed. The wide margin was achieved in this group of patients. Because MA could be related to other factors such as tumor boundary recognition in preoperative imaging, rather than navigation technology only, we did not compare MA between the two groups.

The data were reviewed for statistical analysis. Descriptive statistics were calculated and *t*-test analyses were performed to compare data in the two groups. The data were evaluated for significant differences in the mean and 95% confidence interval (95% CI). Values of *P* < 0.05 were considered statistically significant. The Kaplan–Meier survival analysis was used for the oncological result detection. IBM SPSS Statistics for Windows, Version 19.0 software (IBM Corp., Armonk, NY) was used for all statistical analyses.

## Results

All resections were performed for bone sarcomas around the knee in the navigation and freehand groups by the same three groups of experienced orthopedic oncology surgeons. Navigation was conducted successfully in all 39 cases. The length of the planned resection was measured by the navigation planning system in the navigation group or on the imaging working station in the freehand group. The length of the specimen in millimeters was measured with a ruler after splitting in the coronal direction using an electronic saw, except for four cases treated with recycled frozen autografts that were measured without splitting. In the navigation group, the length of the planned resection or specimen was measured between the two centers of the cutting planes, while in the freehand group, the length was measured from the center of the joint line to the center of the cutting plane in both the planned resection and the specimen. After surgery, the margin was checked by pathologists, and both groups were confirmed to have wide margins at the bone cutting site and soft tissue part.

The resection length in the navigation group was 180 ± 48 mm (85–282 mm), while those in the freehand group was 175 ± 43 mm (90–330 mm). The LD between the postoperative specimen and preoperative planned length was detected. In the navigation group, the LD was 0.5 ± 2.5 mm (range, − 5 to 5 mm), while in the freehand group, the LD was 3.4 ± 9.6 mm (range, − 20 to 29 mm), with a significant difference (*P* < 0.01). For the absolute value analysis, the LD absolute value was 2.0 ± 1.6 mm in the navigation group and 8.3 ± 6.0 mm in the freehand group, with a significant difference (*P* < 0.01).

The achieved and planned bony margins were detected, and the MA was calculated. The MA was 0.3 ± 1.5 mm (range, − 3 to 3 mm) and the MA absolute value was 1.1 ± 1.0 mm (Table 2).

**Table 2:** The comparison of two groups

	Navigation group	Control group	
Resection length	180 ± 48 mm (85–282 mm)	175 ± 43 mm (90–330 mm)	*P* > 0.05
LD	0.5 ± 2.5 mm (− 5 to 5 mm)	3.4 ± 9.6 mm (− 20 to 29 mm)	*P* < 0.01
LD absolute value	2.0 ± 1.6 mm	8.3 ± 6.0 mm	*P* < 0.01
MA	0.3 ± 1.5 mm (− 3 to 3 mm)		
MA absolute value	1.1 ± 1.0 mm (0–3 mm)		

*LD* the length difference, *MA* the margin accuracy

**Fig. 8:**
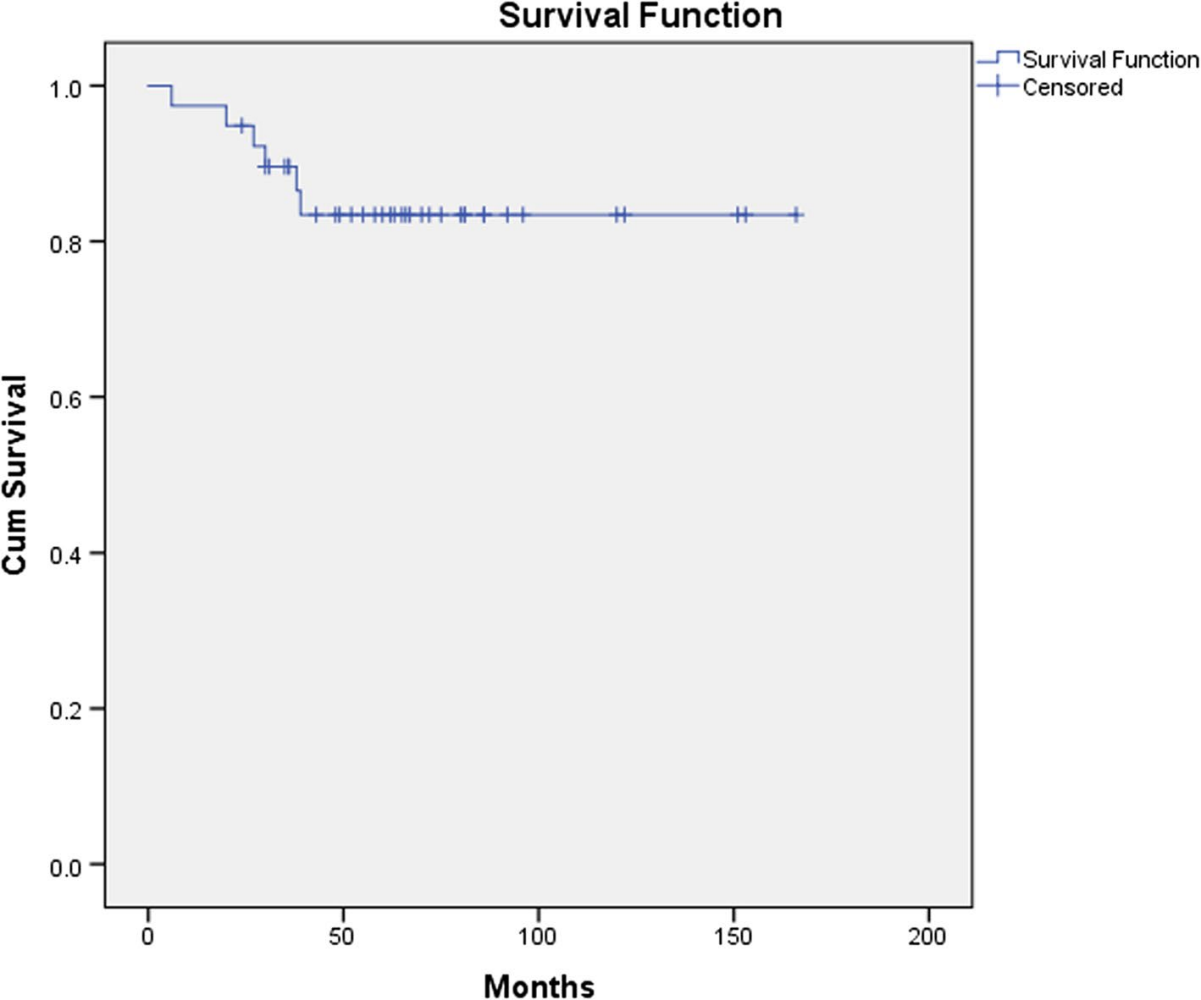
The Kaplan–Meier survival analysis for the patients of the navigation group

All the 39 patients in the navigation group were followed up 6–166 months, the average follow-up was 86.5 months. At the end of the follow-up, six patients died of disease. Thirty-one patients were continuously disease free. Two patients were alive with disease. The 5 years survival rate was 83.4% (Fig. 8). The local soft tissue recurrence was detected in five patients.

## Discussion

Computer-assisted navigation has become a useful and accurate tool for bone tumor surgeons. The technology has been applied in many bone tumor surgeries, including pelvic and sacral tumor resection [2, 14], joint-sparing surgery [5, 6], extremity tumor resection [8], spinal tumor removal [15], and minimal invasive approach for osteoid osteoma [16]. The advantages of using navigation include acquisition of a negative surgical margin and reduction in surgical trauma, which have oncological and functional benefits for patients. The resection plan is at least 10 mm away from the tumor when navigation is conducted for the safety to obtain a negative margin [6]. Meanwhile, more time carrying out with the planning and intraoperative procedure when computer-assisted navigation is applied. In the present study, we included cases of intercalary resection in the distal femur and proximal tibia with navigation. The purpose of navigation use in early cases was to make the resection more precise to match the length of a custom-made prosthesis. The early clinical benefit led us to use this technology when precise resection was attempted around the knee.

The accuracy of computer-assisted navigation varies among different bone tumor procedures. Some studies have focused on the accuracy of navigation in bone tumor models with a control group. Cartiaux et al. [10] conducted an experimental study using a pelvic bone model. Twenty-three operators were asked to perform cutting of a tumor. The mean difference was 2.8 mm when navigation was used, compared with 11.2 mm for a freehand cutting procedure. The location accuracy of the cut planes with respect to the target planes was significantly improved by navigation use. In a study by Sternheim et al. [11], 126 navigated cuts were performed by orthopedic oncologists, and another 126 non-navigated cuts were carried out by the same surgeons. None of the 27 navigated resections violated the tumor while 6 of the 27 non-navigated resections (22%) cut into the tumor. The mean distance from the planned bone cuts to the actual entry points into bone was 1.5 ± 1.4 mm for all navigation users. In a cadaver study [9], the authors compared computer-assisted surgery (CAS), patient-specific instrumentation (PSI), and freehand resection. The freehand group was significantly less accurate than the CAS, PSI, and CAS + PSI groups. The mean location accuracy was 9.2 ± 3.3 mm in the freehand group, compared with 3.6 ± 2.1 mm in the CAS group. Meanwhile, the location accuracy was 1.9 ± 1.1 mm in the PSI group and 2.0 ± 1.0 mm in the CAS + PSI group. This cadaveric study indicated that PSI was the most accurate method for assistance with tumor resection. In clinical practice, the soft tissue outside bone must be taken into consideration when performing bone sarcoma surgery. An adequate margin for both bone and soft tissue should always be the first consideration for better survival [14]. Thus, navigation in clinical patients is more difficult than that in experimental or cadaveric studies. In a study by Ieguchi et al. [12], the authors included 16 patients for analysis, and found that the mean difference was 0–4 mm between the planned margin and the postoperative CT image or excised histologic specimen. In a study by Ritacco et al. [13], 28 cases were included and the global mean of the quantitative data was 2.52 ± 2.32 mm between the programmed osteotomy and the specimen.

In our study, there were two cutting sites at the bone in the navigation group, but only one cutting site in the control group, because we did not have a group of patients with exactly the same surgery. The patients were treated by the same three groups of surgeons to balance the experience of the doctors, because experience is another factor for accurate cutting [17]. In the control group, there were three times more patients than in the navigation group for the numbers of both the distal femur and proximal tibia cases. Our data showed that bone cutting with navigation was more accurate than freehand cutting. The mean LD (absolute value) was 2.0 mm (95% CI, 0.4–3.6 mm) in the navigation group, compared with 8.3 mm (95% CI, 2.3–14.3 mm) in the freehand group. The improved accuracy with navigation was similar to the findings in previous studies [7–11]. As a clinical study, to the best of our knowledge, we set a control group for the first time and provided data on the accuracy that can be achieved by experienced bone tumor surgeons with the help of computer-assisted navigation compared with freehand resection around the knee.

The accuracy of tumor resection in the extremities depends on confirmation of the resection plane and the instrument used for osteotomy. In a previous study [18], the achieved and planned lengths differed significantly in the distal femur and proximal tibia resections performed freehand. Therefore, the present study compared the accuracy when navigation or freehand was used. In freehand resection, a landmark should be chosen preoperatively, and this landmark should be easy to expose during surgery for intraoperative use. Usually, the knee joint line is the landmark for the distal femur and proximal tibia resection. The cutting plane is determined by ruler measurement in freehand resection. In the present study, the surgeons used a wire saw to cut the bone in both groups. Navigation can help to confirm the cutting plane and guide the direction of the wire saw, which is the reason why the navigation group is more precise.

New technologies are constantly emerging in the clinical ambit. Navigation has been widely used in orthopedic surgery. Some studies on computer-assisted navigation have demonstrated good results in multiplanar osteotomy, especially in the pelvic region [2, 4, 14]. Our study provides new evidence for accuracy when extremity tumor resection was performed under navigation. We can thus add our evidence for uniplanar resection to the literature. The LD in the navigation group was superior to that in the freehand group. The MA was acceptable with navigation use since all the MAs were less than 1 cm, which could guarantee the negative margin when we designed at least 1 cm distance from the tumor boundary. The reconstruction methods varied among different procedures. When a custom-made or 3D-printed prosthesis was designed for use after resection, higher accuracy was required during tumor resection. Therefore, navigation can provide great benefits in these circumstances. According to an experimental study [9], PSI may be a method to achieve better accuracy than navigation only in the future. However, in clinical practice, the position for PSI is more difficult than that in an experimental study because of the soft tissue surrounding the bone and the tumor. New imaging technologies such as O-arm CT [19] or cone-beam CT [20, 21] have been used for navigation in recent years, and have also shown benefits for patients.

In recent years, robot-assisted surgery is conducted for bone tumor resection or ablation [22, 23]. The stable robot arm can help to confirm the cutting plane and direction. But the cutting tool is still a challenge when used in the robot arm to perform the osteotomy. The 3D printing technology has been conducted in the reconstruction of bone defect after tumor resection, which is more precise when navigation is used in the surgery [24].

The present study has several limitations. First, we did not have a control group undergoing the same surgical procedure as the navigation group. We need more time to carry out the preoperative planning and register images to the patient. However, we cannot compare the operation times because of the different kinds of procedures in the two groups. Second, our study focused on the accuracy of cutting length, not comparing the different reconstruction methods (prosthesis, allograft and recycle bone) because of the small numbers. Third, the time span was very long because cases were included from September 2008 to November 2017, and during this long period, the increasing experience of surgeons in using the navigation may have an impact on the outcome.

In conclusion, better accuracy can be achieved when navigation is conducted for bone sarcoma resection around the knee. Computer-assisted navigation can play an important role in limb salvage surgery when precise resection is required.
